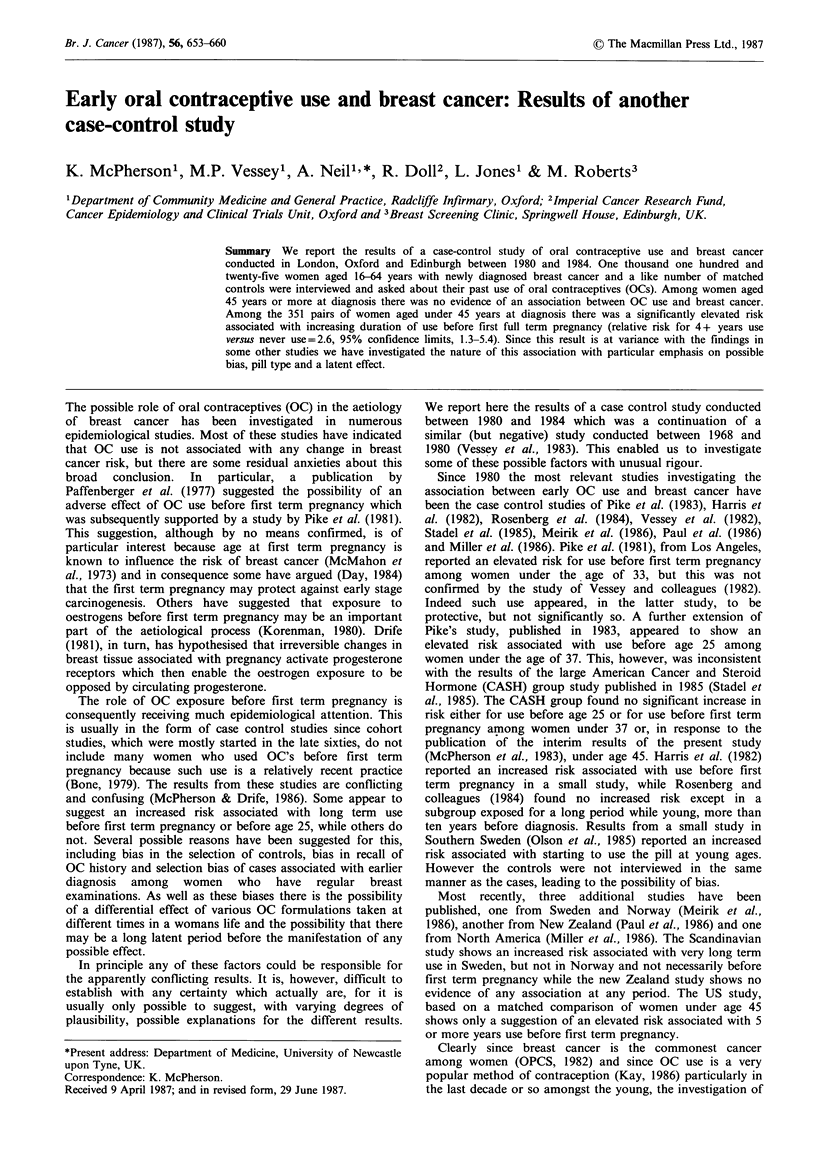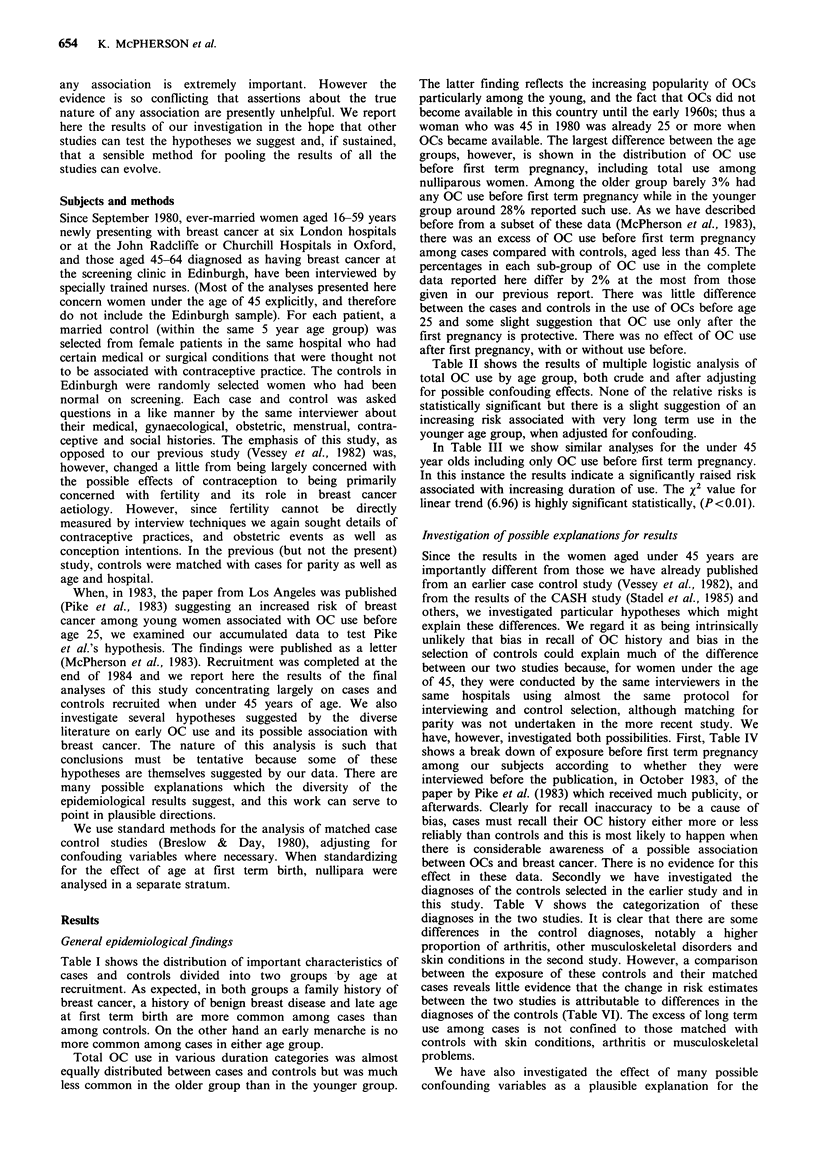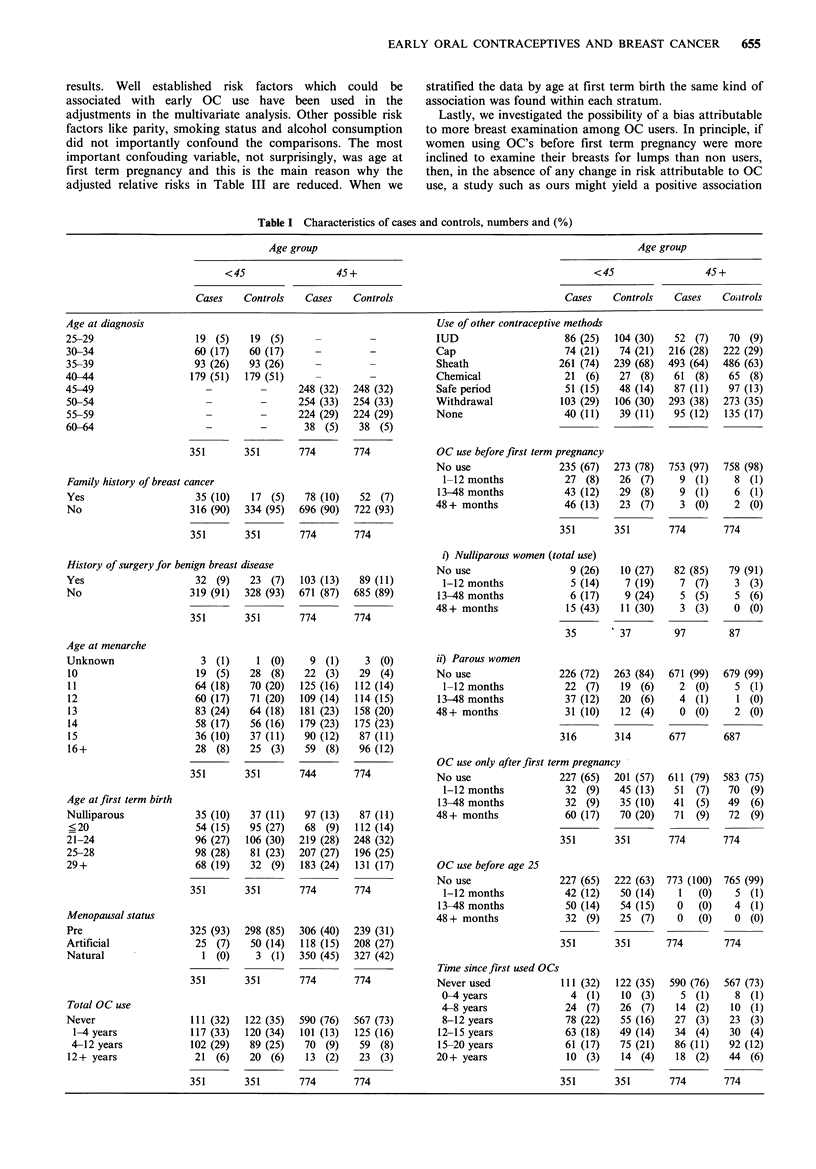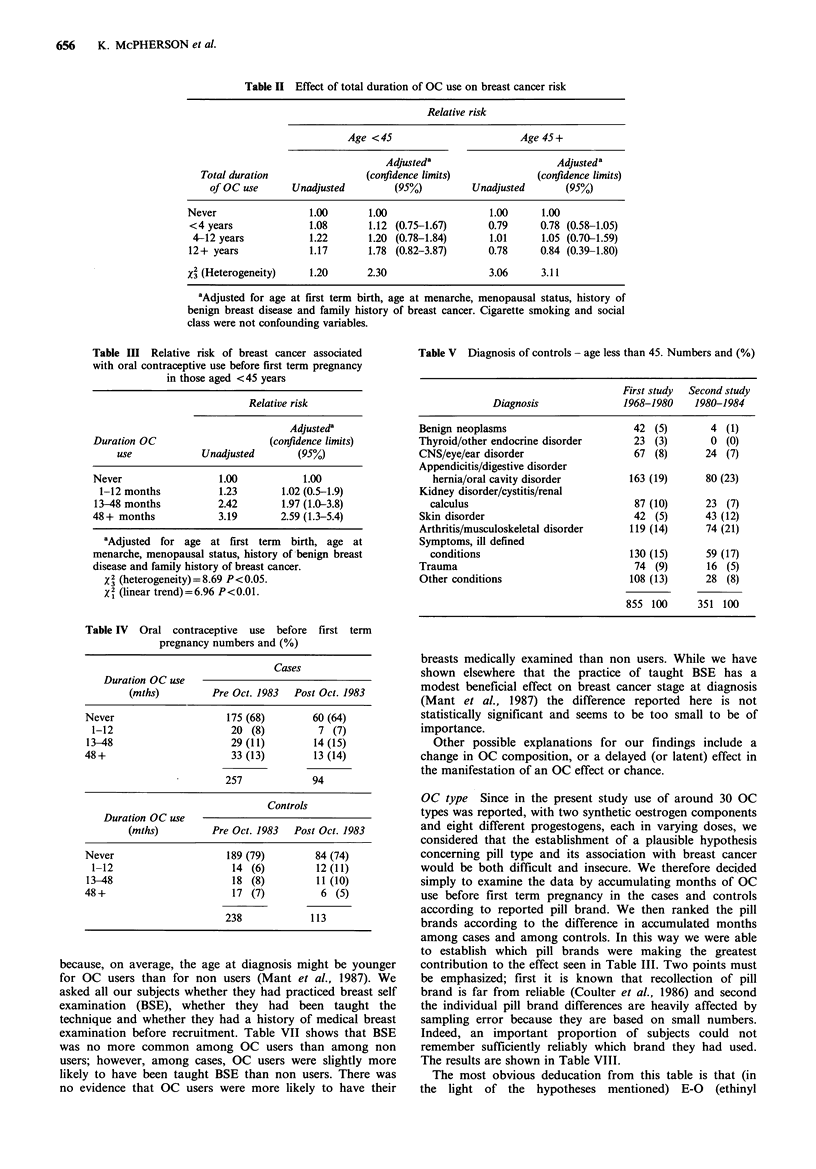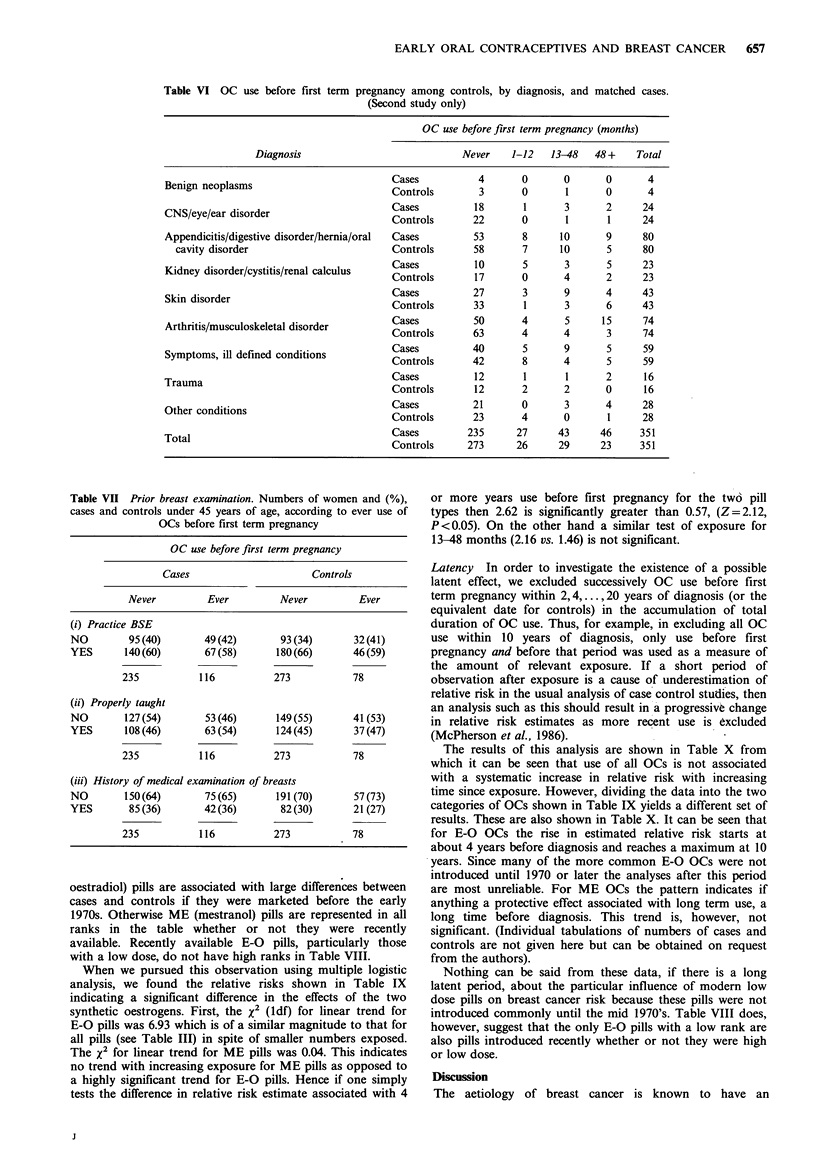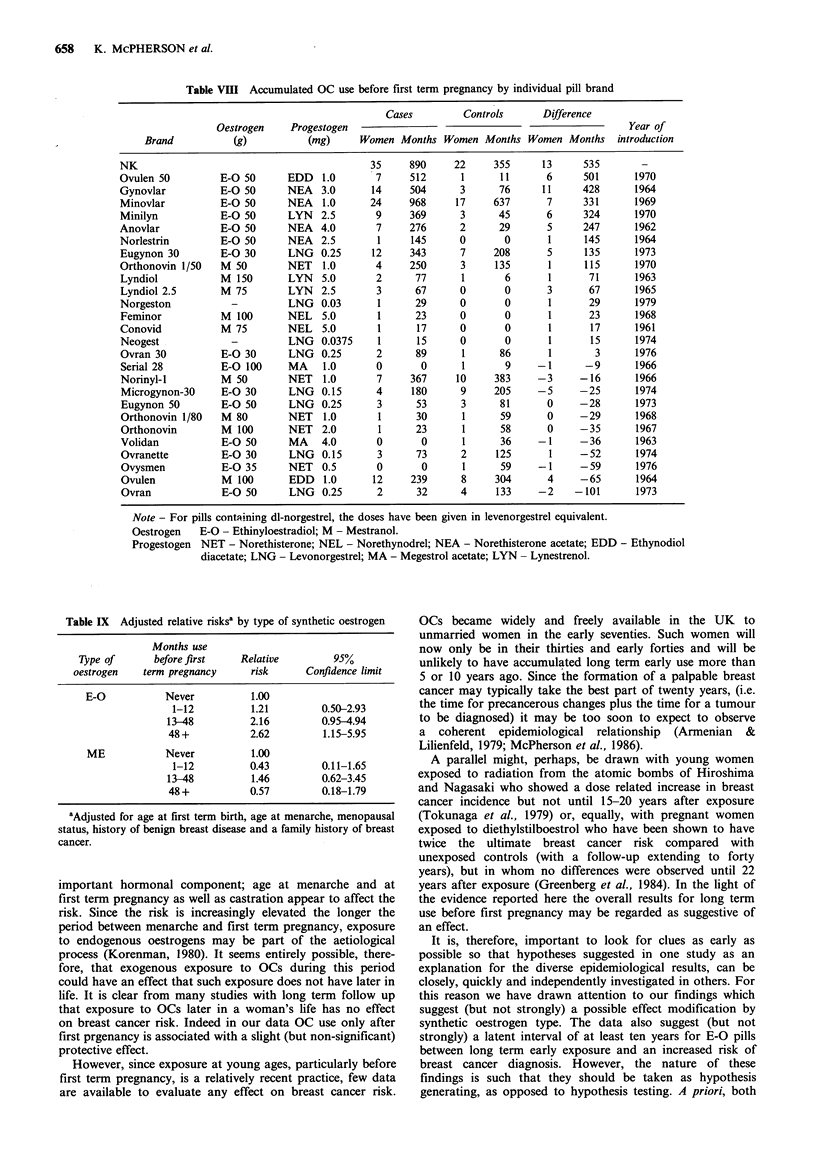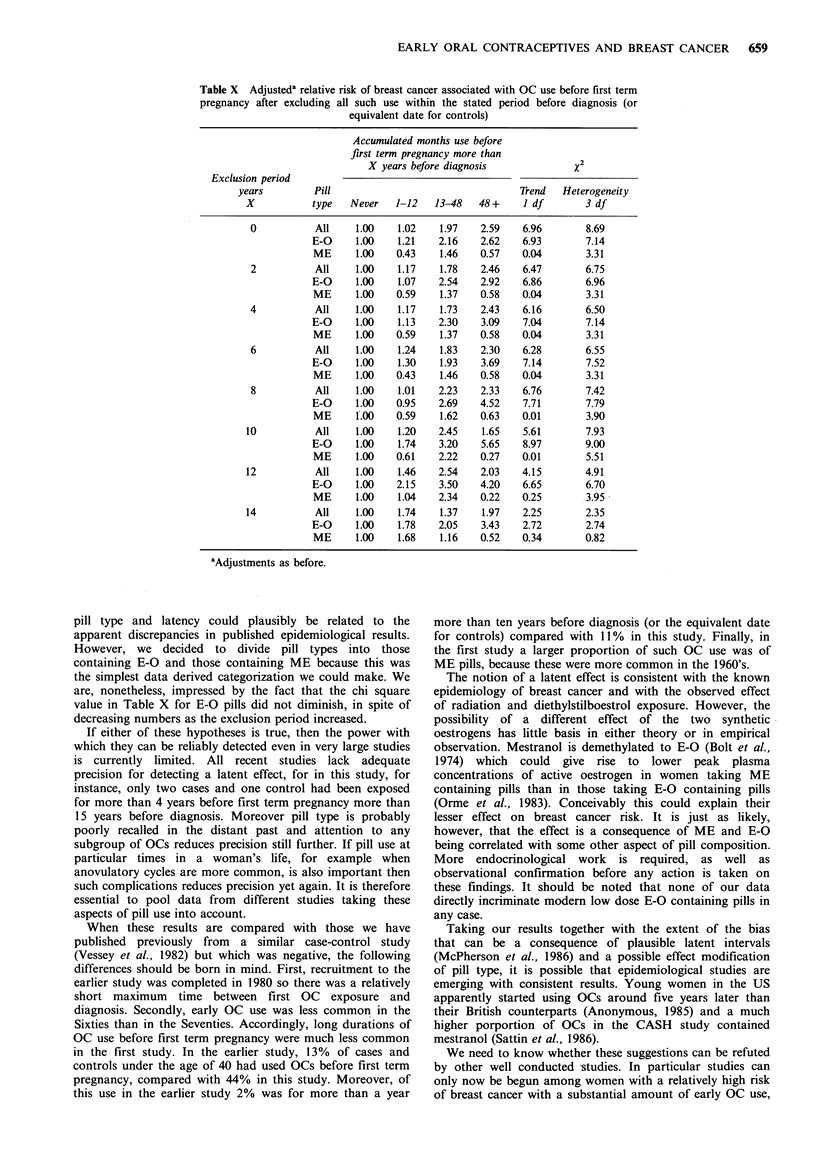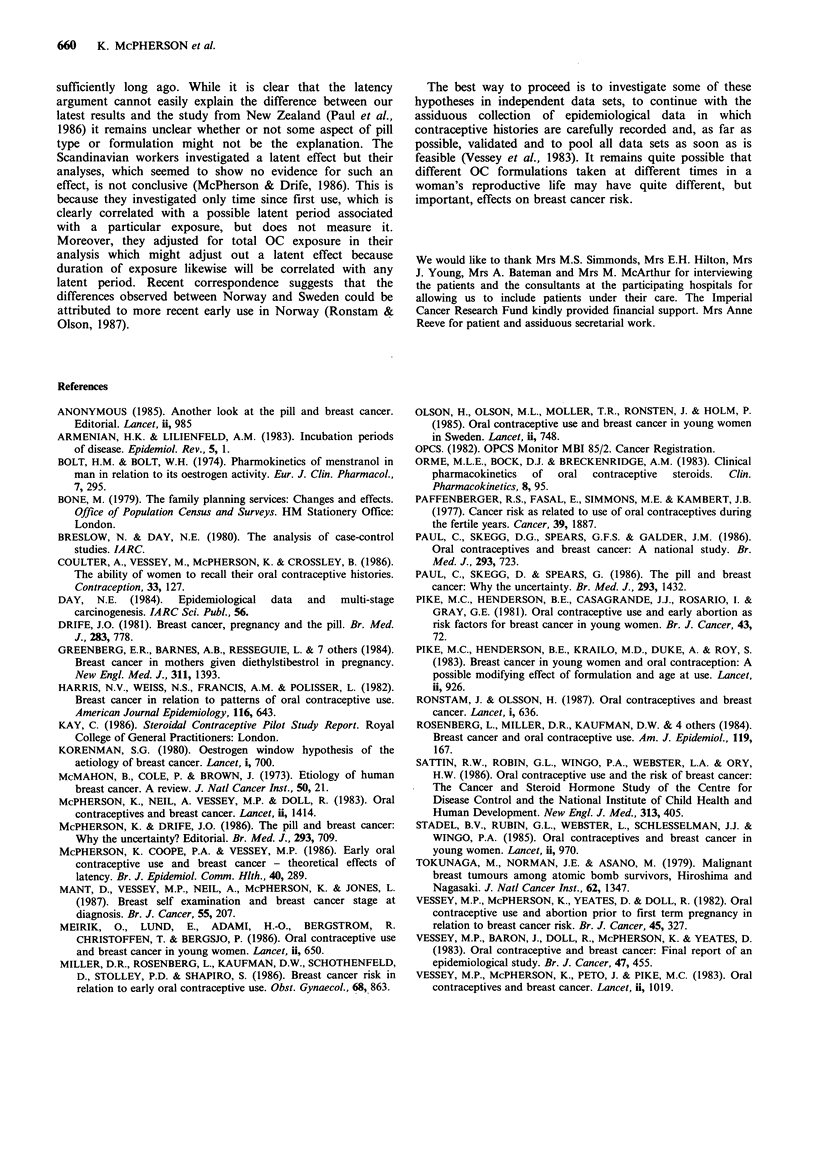# Early oral contraceptive use and breast cancer: results of another case-control study.

**DOI:** 10.1038/bjc.1987.261

**Published:** 1987-11

**Authors:** K. McPherson, M. P. Vessey, A. Neil, R. Doll, L. Jones, M. Roberts

**Affiliations:** Department of Community Medicine and General Practice, Radcliffe Infirmary, Oxford, UK.

## Abstract

We report the results of a case-control study of oral contraceptive use and breast cancer conducted in London, Oxford and Edinburgh between 1980 and 1984. One thousand one hundred and twenty-five women aged 16-64 years with newly diagnosed breast cancer and a like number of matched controls were interviewed and asked about their past due use of oral contraceptives (OCs). Among women aged 45 years or more at diagnosis there was no evidence of an association between OC use and breast cancer. Among the 351 pairs of women aged under 45 years at diagnosis there was a significantly elevated risk associated with increasing duration of use before first full term pregnancy (relative risk for 4+ years use versus never use = 2.6, 95% confidence limits, 1.3-5.4). Since this result is at variance with the findings in some other studies we have investigated the nature of this association with particular emphasis on possible bias, pill type and a latent effect.


Br. J. Cancer (1987), 56, 653–660 © The Macmillan Press Ltd., 1987

### Early oral contraceptive use and breast cancer: Results of another case-control study

K. McPherson[1], M.P. Vessey[1], A. Neil[1,*], R. Doll[2], L. Jones[1] & M. Roberts[3]

[1]*Department of Community Medicine and General Practice, Radcliffe Infirmary, Oxford;* [2]*Imperial Cancer Research Fund, Cancer Epidemiology and Clinical Trials Unit, Oxford and* [3]*Breast Screening Clinic, Springwell House, Edinburgh, UK.*

**Summary** We report the results of a case-control study of oral contraceptive use and breast cancer conducted in London, Oxford and Edinburgh between 1980 and 1984. One thousand one hundred and twenty-five women aged 16–64 years with newly diagnosed breast cancer and a like number of matched controls were interviewed and asked about their past use of oral contraceptives (OCs). Among women aged 45 years or more at diagnosis there was no evidence of an association between OC use and breast cancer. Among the 351 pairs of women aged under 45 years at diagnosis there was a significantly elevated risk associated with increasing duration of use before first full term pregnancy (relative risk for 4+ years use *versus* never use=2.6, 95% confidence limits, 1.3–5.4). Since this result is at variance with the findings in some other studies we have investigated the nature of this association with particular emphasis on possible bias, pill type and a latent effect.

The possible role of oral contraceptives (OC) in the aetiology of breast cancer has been investigated in numerous epidemiological studies. Most of these studies have indicated that OC use is not associated with any change in breast cancer risk, but there are some residual anxieties about this broad conclusion. In particular, a publication by Paffenberger *et al.* (1977) suggested the possibility of an adverse effect of OC use before first term pregnancy which was subsequently supported by a study by Pike *et al.* (1981). This suggestion, although by no means confirmed, is of particular interest because age at first term pregnancy is known to influence the risk of breast cancer (McMahon *et al.*, 1973) and in consequence some have argued (Day, 1984) that the first term pregnancy may protect against early stage carcinogenesis. Others have suggested that exposure to oestrogens before first term pregnancy may be an important part of the aetiological process (Korenman, 1980). Drife (1981), in turn, has hypothesised that irreversible changes in breast tissue associated with pregnancy activate progesterone receptors which then enable the oestrogen exposure to be opposed by circulating progesterone.

The role of OC exposure before first term pregnancy is consequently receiving much epidemiological attention. This is usually in the form of case control studies since cohort studies, which were mostly started in the late sixties, do not include many women who used OC's before first term pregnancy because such use is a relatively recent practice (Bone, 1979). The results from these studies are conflicting and confusing (McPherson & Drife, 1986). Some appear to suggest an increased risk associated with long term use before first term pregnancy or before age 25, while others do not. Several possible reasons have been suggested for this, including bias in the selection of controls, bias in recall of OC history and selection bias of cases associated with earlier diagnosis among women who have regular breast examinations. As well as these biases there is the possibility of a differential effect of various OC formulations taken at different times in a womans life and the possibility that there may be a long latent period before the manifestation of any possible effect.

In principle any of these factors could be responsible for the apparently conflicting results. It is, however, difficult to establish with any certainty which actually are, for it is usually only possible to suggest, with varying degrees of plausibility, possible explanations for the different results. We report here the results of a case control study conducted between 1980 and 1984 which was a continuation of a similar (but negative) study conducted between 1968 and 1980 (Vessey *et al.*, 1983). This enabled us to investigate some of these possible factors with unusual rigour.

Since 1980 the most relevant studies investigating the association between early OC use and breast cancer have been the case control studies of Pike *et al.* (1983), Harris *et al.* (1982), Rosenberg *et al.* (1984), Vessey *et al.* (1982), Stadel *et al.* (1985), Meirik *et al.* (1986), Paul *et al.* (1986) and Miller *et al.* (1986). Pike *et al.* (1981), from Los Angeles, reported an elevated risk for use before first term pregnancy among women under the age of 33, but this was not confirmed by the study of Vessey and colleagues (1982). Indeed such use appeared, in the latter study, to be protective, but not significantly so. A further extension of Pike's study, published in 1983, appeared to show an elevated risk associated with use before age 25 among women under the age of 37. This, however, was inconsistent with the results of the large American Cancer and Steroid Hormone (CASH) group study published in 1985 (Stadel *et al.*, 1985). The CASH group found no significant increase in risk either for use before age 25 or for use before first term pregnancy among women under 37 or, in response to the publication of the interim results of the present study (McPherson *et al.*, 1983), under age 45. Harris *et al.* (1982) reported an increased risk associated with use before first term pregnancy in a small study, while Rosenberg and colleagues (1984) found no increased risk except in a subgroup exposed for a long period while young, more than ten years before diagnosis. Results from a small study in Southern Sweden (Olson *et al.*, 1985) reported an increased risk associated with starting to use the pill at young ages. However the controls were not interviewed in the same manner as the cases, leading to the possibility of bias.

Most recently, three additional studies have been published, one from Sweden and Norway (Meirik *et al.*, 1986), another from New Zealand (Paul *et al.*, 1986) and one from North America (Miller *et al.*, 1986). The Scandinavian study shows an increased risk associated with very long term use in Sweden, but not in Norway and not necessarily before first term pregnancy while the new Zealand study shows no evidence of any association at any period. The US study, based on a matched comparison of women under age 45 shows only a suggestion of an elevated risk associated with 5 or more years use before first term pregnancy.

Clearly since breast cancer is the commonest cancer among women (OPCS, 1982) and since OC use is a very popular method of contraception (Kay, 1986) particularly in the last decade or so amongst the young, the investigation of

*Present address: Department of Medicine, University of Newcastle upon Tyne, UK.
Correspondence: K. McPherson.
Received 9 April 1987; and in revised form, 29 June 1987.

any association is extremely important. However the evidence is so conflicting that assertions about the true nature of any association are presently unhelpful. We report here the results of our investigation in the hope that other studies can test the hypotheses we suggest and, if sustained, that a sensible method for pooling the results of all the studies can evolve.

### Subjects and methods

Since September 1980, ever-married women aged 16–59 years newly presenting with breast cancer at six London hospitals or at the John Radcliffe or Churchill Hospitals in Oxford, and those aged 45–64 diagnosed as having breast cancer at the screening clinic in Edinburgh, have been interviewed by specially trained nurses. (Most of the analyses presented here concern women under the age of 45 explicitly, and therefore do not include the Edinburgh sample). For each patient, a married control (within the same 5 year age group) was selected from female patients in the same hospital who had certain medical or surgical conditions that were thought not to be associated with contraceptive practice. The controls in Edinburgh were randomly selected women who had been normal on screening. Each case and control was asked questions in a like manner by the same interviewer about their medical, gynaecological, obstetric, menstrual, contraceptive and social histories. The emphasis of this study, as opposed to our previous study (Vessey et al., 1982) was, however, changed a little from being largely concerned with the possible effects of contraception to being primarily concerned with fertility and its role in breast cancer aetiology. However, since fertility cannot be directly measured by interview techniques we again sought details of contraceptive practices, and obstetric events as well as conception intentions. In the previous (but not the present) study, controls were matched with cases for parity as well as age and hospital.

When, in 1983, the paper from Los Angeles was published (Pike et al., 1983) suggesting an increased risk of breast cancer among young women associated with OC use before age 25, we examined our accumulated data to test Pike et al.'s hypothesis. The findings were published as a letter (McPherson et al., 1983). Recruitment was completed at the end of 1984 and we report here the results of the final analyses of this study concentrating largely on cases and controls recruited when under 45 years of age. We also investigate several hypotheses suggested by the diverse literature on early OC use and its possible association with breast cancer. The nature of this analysis is such that conclusions must be tentative because some of these hypotheses are themselves suggested by our data. There are many possible explanations which the diversity of the epidemiological results suggest, and this work can serve to point in plausible directions.

We use standard methods for the analysis of matched case control studies (Breslow & Day, 1980), adjusting for confouding variables where necessary. When standardizing for the effect of age at first term birth, nullipara were analysed in a separate stratum.

### Results

### *General epidemiological findings*

Table I shows the distribution of important characteristics of cases and controls divided into two groups by age at recruitment. As expected, in both groups a family history of breast cancer, a history of benign breast disease and late age at first term birth are more common among cases than among controls. On the other hand an early menarche is no more common among cases in either age group.

Total OC use in various duration categories was almost equally distributed between cases and controls but was much less common in the older group than in the younger group. The latter finding reflects the increasing popularity of OCs particularly among the young, and the fact that OCs did not become available in this country until the early 1960s; thus a woman who was 45 in 1980 was already 25 or more when OCs became available. The largest difference between the age groups, however, is shown in the distribution of OC use before first term pregnancy, including total use among nulliparous women. Among the older group barely 3% had any OC use before first term pregnancy while in the younger group around 28% reported such use. As we have described before from a subset of these data (McPherson et al., 1983), there was an excess of OC use before first term pregnancy among cases compared with controls, aged less than 45. The percentages in each sub-group of OC use in the complete data reported here differ by 2% at the most from those given in our previous report. There was little difference between the cases and controls in the use of OCs before age 25 and some slight suggestion that OC use only after the first pregnancy is protective. There was no effect of OC use after first pregnancy, with or without use before.

Table II shows the results of multiple logistic analysis of total OC use by age group, both crude and after adjusting for possible confouding effects. None of the relative risks is statistically significant but there is a slight suggestion of an increasing risk associated with very long term use in the younger age group, when adjusted for confouding.

In Table III we show similar analyses for the under 45 year olds including only OC use before first term pregnancy. In this instance the results indicate a significantly raised risk associated with increasing duration of use. The $\chi^2$ value for linear trend (6.96) is highly significant statistically, ($P<0.01$).

### *Investigation of possible explanations for results*

Since the results in the women aged under 45 years are importantly different from those we have already published from an earlier case control study (Vessey et al., 1982), and from the results of the CASH study (Stadel et al., 1985) and others, we investigated particular hypotheses which might explain these differences. We regard it as being intrinsically unlikely that bias in recall of OC history and bias in the selection of controls could explain much of the difference between our two studies because, for women under the age of 45, they were conducted by the same interviewers in the same hospitals using almost the same protocol for interviewing and control selection, although matching for parity was not undertaken in the more recent study. We have, however, investigated both possibilities. First, Table IV shows a break down of exposure before first term pregnancy among our subjects according to whether they were interviewed before the publication, in October 1983, of the paper by Pike et al. (1983) which received much publicity, or afterwards. Clearly for recall inaccuracy to be a cause of bias, cases must recall their OC history either more or less reliably than controls and this is most likely to happen when there is considerable awareness of a possible association between OCs and breast cancer. There is no evidence for this effect in these data. Secondly we have investigated the diagnoses of the controls selected in the earlier study and in this study. Table V shows the categorization of these diagnoses in the two studies. It is clear that there are some differences in the control diagnoses, notably a higher proportion of arthritis, other musculoskeletal disorders and skin conditions in the second study. However, a comparison between the exposure of these controls and their matched cases reveals little evidence that the change in risk estimates between the two studies is attributable to differences in the diagnoses of the controls (Table VI). The excess of long term use among cases is not confined to those matched with controls with skin conditions, arthritis or musculoskeletal problems.

We have also investigated the effect of many possible confounding variables as a plausible explanation for the results. Well established risk factors which could be associated with early OC use have been used in the adjustments in the multivariate analysis. Other possible risk factors like parity, smoking status and alcohol consumption did not importantly confound the comparisons. The most important confouding variable, not surprisingly, was age at first term pregnancy and this is the main reason why the adjusted relative risks in Table III are reduced. When we stratified the data by age at first term birth the same kind of association was found within each stratum.

Lastly, we investigated the possibility of a bias attributable to more breast examination among OC users. In principle, if women using OC's before first term pregnancy were more inclined to examine their breasts for lumps than non users, then, in the absence of any change in risk attributable to OC use, a study such as ours might yield a positive association

**Table I** Characteristics of cases and controls, numbers and (%)

| | *Age group* | | | |
|---|---|---|---|---|
| | *<45* | | *45+* | |
| | *Cases* | *Controls* | *Cases* | *Controls* |
| *Age at diagnosis* | | | | |
| 25–29 | 19 (5) | 19 (5) | – | – |
| 30–34 | 60 (17) | 60 (17) | – | – |
| 35–39 | 93 (26) | 93 (26) | – | – |
| 40–44 | 179 (51) | 179 (51) | – | – |
| 45–49 | – | – | 248 (32) | 248 (32) |
| 50–54 | – | – | 254 (33) | 254 (33) |
| 55–59 | – | – | 224 (29) | 224 (29) |
| 60–64 | – | – | 38 (5) | 38 (5) |
| | 351 | 351 | 774 | 774 |
| *Family history of breast cancer* | | | | |
| Yes | 35 (10) | 17 (5) | 78 (10) | 52 (7) |
| No | 316 (90) | 334 (95) | 696 (90) | 722 (93) |
| | 351 | 351 | 774 | 774 |
| *History of surgery for benign breast disease* | | | | |
| Yes | 32 (9) | 23 (7) | 103 (13) | 89 (11) |
| No | 319 (91) | 328 (93) | 671 (87) | 685 (89) |
| | 351 | 351 | 774 | 774 |
| *Age at menarche* | | | | |
| Unknown | 3 (1) | 1 (0) | 9 (1) | 3 (0) |
| 10 | 19 (5) | 28 (8) | 22 (3) | 29 (4) |
| 11 | 64 (18) | 70 (20) | 125 (16) | 112 (14) |
| 12 | 60 (17) | 71 (20) | 109 (14) | 114 (15) |
| 13 | 83 (24) | 64 (18) | 181 (23) | 158 (20) |
| 14 | 58 (17) | 56 (16) | 179 (23) | 175 (23) |
| 15 | 36 (10) | 37 (11) | 90 (12) | 87 (11) |
| 16+ | 28 (8) | 25 (3) | 59 (8) | 96 (12) |
| | 351 | 351 | 744 | 774 |
| *Age at first term birth* | | | | |
| Nulliparous | 35 (10) | 37 (11) | 97 (13) | 87 (11) |
| ≦20 | 54 (15) | 95 (27) | 68 (9) | 112 (14) |
| 21-24 | 96 (27) | 106 (30) | 219 (28) | 248 (32) |
| 25–28 | 98 (28) | 81 (23) | 207 (27) | 196 (25) |
| 29+ | 68 (19) | 32 (9) | 183 (24) | 131 (17) |
| | 351 | 351 | 774 | 774 |
| *Menopausal status* | | | | |
| Pre | 325 (93) | 298 (85) | 306 (40) | 239 (31) |
| Artificial | 25 (7) | 50 (14) | 118 (15) | 208 (27) |
| Natural | 1 (0) | 3 (1) | 350 (45) | 327 (42) |
| | 351 | 351 | 774 | 774 |
| *Total OC use* | | | | |
| Never | 111 (32) | 122 (35) | 590 (76) | 567 (73) |
| 1–4 years | 117 (33) | 120 (34) | 101 (13) | 125 (16) |
| 4–12 years | 102 (29) | 89 (25) | 70 (9) | 59 (8) |
| 12+ years | 21 (6) | 20 (6) | 13 (2) | 23 (3) |
| | 351 | 351 | 774 | 774 |

| | *Age group* | | | |
|---|---|---|---|---|
| | *<45* | | *45+* | |
| | *Cases* | *Controls* | *Cases* | *Controls* |
| *Use of other contraceptive methods* | | | | |
| IUD | 86 (25) | 104 (30) | 52 (7) | 70 (9) |
| Cap | 74 (21) | 74 (21) | 216 (28) | 222 (29) |
| Sheath | 261 (74) | 239 (68) | 493 (64) | 486 (63) |
| Chemical | 21 (6) | 27 (8) | 61 (8) | 65 (8) |
| Safe period | 51 (15) | 48 (14) | 87 (11) | 97 (13) |
| Withdrawal | 103 (29) | 106 (30) | 293 (38) | 273 (35) |
| None | 40 (11) | 39 (11) | 95 (12) | 135 (17) |
| *OC use before first term pregnancy* | | | | |
| No use | 235 (67) | 273 (78) | 753 (97) | 758 (98) |
| 1–12 months | 27 (8) | 26 (7) | 9 (1) | 8 (1) |
| 13–48 months | 43 (12) | 29 (8) | 9 (1) | 6 (1) |
| 48+ months | 46 (13) | 23 (7) | 3 (0) | 2 (0) |
| | 351 | 351 | 774 | 774 |
| *i) Nulliparous women (total use)* | | | | |
| No use | 9 (26) | 10 (27) | 82 (85) | 79 (91) |
| 1–12 months | 5 (14) | 7 (19) | 7 (7) | 3 (3) |
| 13–48 months | 6 (17) | 9 (24) | 5 (5) | 5 (6) |
| 48+ months | 15 (43) | 11 (30) | 3 (3) | 0 (0) |
| | 35 | 37 | 97 | 87 |
| *ii) Parous women* | | | | |
| No use | 226 (72) | 263 (84) | 671 (99) | 679 (99) |
| 1–12 months | 22 (7) | 19 (6) | 2 (0) | 5 (1) |
| 13–48 months | 37 (12) | 20 (6) | 4 (1) | 1 (0) |
| 48+ months | 31 (10) | 12 (4) | 0 (0) | 2 (0) |
| | 316 | 314 | 677 | 687 |
| *OC use only after first term pregnancy* | | | | |
| No use | 227 (65) | 201 (57) | 611 (79) | 583 (75) |
| 1–12 months | 32 (9) | 45 (13) | 51 (7) | 70 (9) |
| 13–48 months | 32 (9) | 35 (10) | 41 (5) | 49 (6) |
| 48+ months | 60 (17) | 70 (20) | 71 (9) | 72 (9) |
| | 351 | 351 | 774 | 774 |
| *OC use before age 25* | | | | |
| No use | 227 (65) | 222 (63) | 773 (100) | 765 (99) |
| 1–12 months | 42 (12) | 50 (14) | 1 (0) | 5 (1) |
| 13–48 months | 50 (14) | 54 (15) | 0 (0) | 4 (1) |
| 48+ months | 32 (9) | 25 (7) | 0 (0) | 0 (0) |
| | 351 | 351 | 774 | 774 |
| *Time since first used OCs* | | | | |
| Never used | 111 (32) | 122 (35) | 590 (76) | 567 (73) |
| 0–4 years | 4 (1) | 10 (3) | 5 (1) | 8 (1) |
| 4–8 years | 24 (7) | 26 (7) | 14 (2) | 10 (1) |
| 8–12 years | 78 (22) | 55 (16) | 27 (3) | 23 (3) |
| 12–15 years | 63 (18) | 49 (14) | 34 (4) | 30 (4) |
| 15–20 years | 61 (17) | 75 (21) | 86 (11) | 92 (12) |
| 20+ years | 10 (3) | 14 (4) | 18 (2) | 44 (6) |
| | 351 | 351 | 774 | 774 |

**Table II** Effect of total duration of OC use on breast cancer risk

| | Relative risk | | | |
|---|---|---|---|---|
| | Age <45 | | Age 45+ | |
| Total duration of OC use | Unadjusted | Adjusted[a] (confidence limits) (95%) | Unadjusted | Adjusted[a] (confidence limits) (95%) |
| Never | 1.00 | 1.00 | 1.00 | 1.00 |
| <4 years | 1.08 | 1.12 (0.75–1.67) | 0.79 | 0.78 (0.58–1.05) |
| 4–12 years | 1.22 | 1.20 (0.78–1.84) | 1.01 | 1.05 (0.70–1.59) |
| 12+ years | 1.17 | 1.78 (0.82–3.87) | 0.78 | 0.84 (0.39–1.80) |
| $\chi^2_3$ (Heterogeneity) | 1.20 | 2.30 | 3.06 | 3.11 |

[a]Adjusted for age at first term birth, age at menarche, menopausal status, history of benign breast disease and family history of breast cancer. Cigarette smoking and social class were not confounding variables.

**Table III** Relative risk of breast cancer associated with oral contraceptive use before first term pregnancy in those aged <45 years

| | Relative risk | |
|---|---|---|
| Duration OC use | Unadjusted | Adjusted[a] (confidence limits) (95%) |
| Never | 1.00 | 1.00 |
| 1–12 months | 1.23 | 1.02 (0.5–1.9) |
| 13–48 months | 2.42 | 1.97 (1.0–3.8) |
| 48+ months | 3.19 | 2.59 (1.3–5.4) |

[a]Adjusted for age at first term birth, age at menarche, menopausal status, history of benign breast disease and family history of breast cancer.
$\chi^2_3$ (heterogeneity) = 8.69 $P<0.05$.
$\chi^2_1$ (linear trend) = 6.96 $P<0.01$.

**Table IV** Oral contraceptive use before first term pregnancy numbers and (%)

| | Cases | |
|---|---|---|
| Duration OC use (mths) | Pre Oct. 1983 | Post Oct. 1983 |
| Never | 175 (68) | 60 (64) |
| 1–12 | 20 (8) | 7 (7) |
| 13–48 | 29 (11) | 14 (15) |
| 48+ | 33 (13) | 13 (14) |
| | 257 | 94 |

| | Controls | |
|---|---|---|
| Duration OC use (mths) | Pre Oct. 1983 | Post Oct. 1983 |
| Never | 189 (79) | 84 (74) |
| 1–12 | 14 (6) | 12 (11) |
| 13–48 | 18 (8) | 11 (10) |
| 48+ | 17 (7) | 6 (5) |
| | 238 | 113 |

because, on average, the age at diagnosis might be younger for OC users than for non users (Mant *et al.*, 1987). We asked all our subjects whether they had practiced breast self examination (BSE), whether they had been taught the technique and whether they had a history of medical breast examination before recruitment. Table VII shows that BSE was no more common among OC users than among non users; however, among cases, OC users were slightly more likely to have been taught BSE than non users. There was no evidence that OC users were more likely to have their breasts medically examined than non users. While we have shown elsewhere that the practice of taught BSE has a modest beneficial effect on breast cancer stage at diagnosis (Mant *et al.*, 1987) the difference reported here is not statistically significant and seems to be too small to be of importance.

**Table V** Diagnosis of controls – age less than 45. Numbers and (%)

| Diagnosis | First study 1968–1980 | Second study 1980–1984 |
|---|---|---|
| Benign neoplasms | 42 (5) | 4 (1) |
| Thyroid/other endocrine disorder | 23 (3) | 0 (0) |
| CNS/eye/ear disorder | 67 (8) | 24 (7) |
| Appendicitis/digestive disorder hernia/oral cavity disorder | 163 (19) | 80 (23) |
| Kidney disorder/cystitis/renal calculus | 87 (10) | 23 (7) |
| Skin disorder | 42 (5) | 43 (12) |
| Arthritis/musculoskeletal disorder | 119 (14) | 74 (21) |
| Symptoms, ill defined conditions | 130 (15) | 59 (17) |
| Trauma | 74 (9) | 16 (5) |
| Other conditions | 108 (13) | 28 (8) |
| | 855 100 | 351 100 |

Other possible explanations for our findings include a change in OC composition, or a delayed (or latent) effect in the manifestation of an OC effect or chance.

*OC type* Since in the present study use of around 30 OC types was reported, with two synthetic oestrogen components and eight different progestogens, each in varying doses, we considered that the establishment of a plausible hypothesis concerning pill type and its association with breast cancer would be both difficult and insecure. We therefore decided simply to examine the data by accumulating months of OC use before first term pregnancy in the cases and controls according to reported pill brand. We then ranked the pill brands according to the difference in accumulated months among cases and among controls. In this way we were able to establish which pill brands were making the greatest contribution to the effect seen in Table III. Two points must be emphasized; first it is known that recollection of pill brand is far from reliable (Coulter *et al.*, 1986) and second the individual pill brand differences are heavily affected by sampling error because they are based on small numbers. Indeed, an important proportion of subjects could not remember sufficiently reliably which brand they had used. The results are shown in Table VIII.

The most obvious deduction from this table is that (in the light of the hypotheses mentioned) E-O (ethinyl

**Table VI** OC use before first term pregnancy among controls, by diagnosis, and matched cases. (Second study only)

| *Diagnosis* | | *OC use before first term pregnancy (months)* Never | 1–12 | 13–48 | 48+ | Total |
|---|---|---|---|---|---|---|
| Benign neoplasms | Cases | 4 | 0 | 0 | 0 | 4 |
| | Controls | 3 | 0 | 1 | 0 | 4 |
| CNS/eye/ear disorder | Cases | 18 | 1 | 3 | 2 | 24 |
| | Controls | 22 | 0 | 1 | 1 | 24 |
| Appendicitis/digestive disorder/hernia/oral cavity disorder | Cases | 53 | 8 | 10 | 9 | 80 |
| | Controls | 58 | 7 | 10 | 5 | 80 |
| Kidney disorder/cystitis/renal calculus | Cases | 10 | 5 | 3 | 5 | 23 |
| | Controls | 17 | 0 | 4 | 2 | 23 |
| Skin disorder | Cases | 27 | 3 | 9 | 4 | 43 |
| | Controls | 33 | 1 | 3 | 6 | 43 |
| Arthritis/musculoskeletal disorder | Cases | 50 | 4 | 5 | 15 | 74 |
| | Controls | 63 | 4 | 4 | 3 | 74 |
| Symptoms, ill defined conditions | Cases | 40 | 5 | 9 | 5 | 59 |
| | Controls | 42 | 8 | 4 | 5 | 59 |
| Trauma | Cases | 12 | 1 | 1 | 2 | 16 |
| | Controls | 12 | 2 | 2 | 0 | 16 |
| Other conditions | Cases | 21 | 0 | 3 | 4 | 28 |
| | Controls | 23 | 4 | 0 | 1 | 28 |
| Total | Cases | 235 | 27 | 43 | 46 | 351 |
| | Controls | 273 | 26 | 29 | 23 | 351 |

**Table VII** *Prior breast examination.* Numbers of women and (%), cases and controls under 45 years of age, according to ever use of OCs before first term pregnancy

| | *OC use before first term pregnancy* Cases: *Never* | Cases: *Ever* | Controls: *Never* | Controls: *Ever* |
|---|---|---|---|---|
| *(i) Practice BSE* | | | | |
| NO | 95 (40) | 49 (42) | 93 (34) | 32 (41) |
| YES | 140 (60) | 67 (58) | 180 (66) | 46 (59) |
| | 235 | 116 | 273 | 78 |
| *(ii) Properly taught* | | | | |
| NO | 127 (54) | 53 (46) | 149 (55) | 41 (53) |
| YES | 108 (46) | 63 (54) | 124 (45) | 37 (47) |
| | 235 | 116 | 273 | 78 |
| *(iii) History of medical examination of breasts* | | | | |
| NO | 150 (64) | 75 (65) | 191 (70) | 57 (73) |
| YES | 85 (36) | 42 (36) | 82 (30) | 21 (27) |
| | 235 | 116 | 273 | 78 |

oestradiol) pills are associated with large differences between cases and controls if they were marketed before the early 1970s. Otherwise ME (mestranol) pills are represented in all ranks in the table whether or not they were recently available. Recently available E-O pills, particularly those with a low dose, do not have high ranks in Table VIII.

When we pursued this observation using multiple logistic analysis, we found the relative risks shown in Table IX indicating a significant difference in the effects of the two synthetic oestrogens. First, the $\chi^2$ (1df) for linear trend for E-O pills was 6.93 which is of a similar magnitude to that for all pills (see Table III) in spite of smaller numbers exposed. The $\chi^2$ for linear trend for ME pills was 0.04. This indicates no trend with increasing exposure for ME pills as opposed to a highly significant trend for E-O pills. Hence if one simply tests the difference in relative risk estimate associated with 4 or more years use before first pregnancy for the two pill types then 2.62 is significantly greater than 0.57, ($Z=2.12$, $P<0.05$). On the other hand a similar test of exposure for 13–48 months (2.16 *vs.* 1.46) is not significant.

*Latency* In order to investigate the existence of a possible latent effect, we excluded successively OC use before first term pregnancy within 2, 4, ..., 20 years of diagnosis (or the equivalent date for controls) in the accumulation of total duration of OC use. Thus, for example, in excluding all OC use within 10 years of diagnosis, only use before first pregnancy *and* before that period was used as a measure of the amount of relevant exposure. If a short period of observation after exposure is a cause of underestimation of relative risk in the usual analysis of case control studies, then an analysis such as this should result in a progressive change in relative risk estimates as more recent use is excluded (McPherson *et al.*, 1986).

The results of this analysis are shown in Table X from which it can be seen that use of all OCs is not associated with a systematic increase in relative risk with increasing time since exposure. However, dividing the data into the two categories of OCs shown in Table IX yields a different set of results. These are also shown in Table X. It can be seen that for E-O OCs the rise in estimated relative risk starts at about 4 years before diagnosis and reaches a maximum at 10 years. Since many of the more common E-O OCs were not introduced until 1970 or later the analyses after this period are most unreliable. For ME OCs the pattern indicates if anything a protective effect associated with long term use, a long time before diagnosis. This trend is, however, not significant. (Individual tabulations of numbers of cases and controls are not given here but can be obtained on request from the authors).

Nothing can be said from these data, if there is a long latent period, about the particular influence of modern low dose pills on breast cancer risk because these pills were not introduced commonly until the mid 1970's. Table VIII does, however, suggest that the only E-O pills with a low rank are also pills introduced recently whether or not they were high or low dose.

### Discussion

The aetiology of breast cancer is known to have an

**Table VIII** Accumulated OC use before first term pregnancy by individual pill brand

| Brand | Oestrogen (g) | Progestogen (mg) | Cases Women | Cases Months | Controls Women | Controls Months | Difference Women | Difference Months | Year of introduction |
|---|---|---|---|---|---|---|---|---|---|
| NK | | | 35 | 890 | 22 | 355 | 13 | 535 | – |
| Ovulen 50 | E-O 50 | EDD 1.0 | 7 | 512 | 1 | 11 | 6 | 501 | 1970 |
| Gynovlar | E-O 50 | NEA 3.0 | 14 | 504 | 3 | 76 | 11 | 428 | 1964 |
| Minovlar | E-O 50 | NEA 1.0 | 24 | 968 | 17 | 637 | 7 | 331 | 1969 |
| Minilyn | E-O 50 | LYN 2.5 | 9 | 369 | 3 | 45 | 6 | 324 | 1970 |
| Anovlar | E-O 50 | NEA 4.0 | 7 | 276 | 2 | 29 | 5 | 247 | 1962 |
| Norlestrin | E-O 50 | NEA 2.5 | 1 | 145 | 0 | 0 | 1 | 145 | 1964 |
| Eugynon 30 | E-O 30 | LNG 0.25 | 12 | 343 | 7 | 208 | 5 | 135 | 1973 |
| Orthonovin 1/50 | M 50 | NET 1.0 | 4 | 250 | 3 | 135 | 1 | 115 | 1970 |
| Lyndiol | M 150 | LYN 5.0 | 2 | 77 | 1 | 6 | 1 | 71 | 1963 |
| Lyndiol 2.5 | M 75 | LYN 2.5 | 3 | 67 | 0 | 0 | 3 | 67 | 1965 |
| Norgeston | – | LNG 0.03 | 1 | 29 | 0 | 0 | 1 | 29 | 1979 |
| Feminor | M 100 | NEL 5.0 | 1 | 23 | 0 | 0 | 1 | 23 | 1968 |
| Conovid | M 75 | NEL 5.0 | 1 | 17 | 0 | 0 | 1 | 17 | 1961 |
| Neogest | – | LNG 0.0375 | 1 | 15 | 0 | 0 | 1 | 15 | 1974 |
| Ovran 30 | E-O 30 | LNG 0.25 | 2 | 89 | 1 | 86 | 1 | 3 | 1976 |
| Serial 28 | E-O 100 | MA 1.0 | 0 | 0 | 1 | 9 | –1 | –9 | 1966 |
| Norinyl-1 | M 50 | NET 1.0 | 7 | 367 | 10 | 383 | –3 | –16 | 1966 |
| Microgynon-30 | E-O 30 | LNG 0.15 | 4 | 180 | 9 | 205 | –5 | –25 | 1974 |
| Eugynon 50 | E-O 50 | LNG 0.25 | 3 | 53 | 3 | 81 | 0 | –28 | 1973 |
| Orthonovin 1/80 | M 80 | NET 1.0 | 1 | 30 | 1 | 59 | 0 | –29 | 1968 |
| Orthonovin | M 100 | NET 2.0 | 1 | 23 | 1 | 58 | 0 | –35 | 1967 |
| Volidan | E-O 50 | MA 4.0 | 0 | 0 | 1 | 36 | –1 | –36 | 1963 |
| Ovranette | E-O 30 | LNG 0.15 | 3 | 73 | 2 | 125 | 1 | –52 | 1974 |
| Ovysmen | E-O 35 | NET 0.5 | 0 | 0 | 1 | 59 | –1 | –59 | 1976 |
| Ovulen | M 100 | EDD 1.0 | 12 | 239 | 8 | 304 | 4 | –65 | 1964 |
| Ovran | E-O 50 | LNG 0.25 | 2 | 32 | 4 | 133 | –2 | –101 | 1973 |

*Note* – For pills containing dl-norgestrel, the doses have been given in levenorgestrel equivalent.
Oestrogen E-O – Ethinyloestradiol; M – Mestranol.
Progestogen NET – Norethisterone; NEL – Norethynodrel; NEA – Norethisterone acetate; EDD – Ethynodiol diacetate; LNG – Levonorgestrel; MA – Megestrol acetate; LYN – Lynestrenol.

**Table IX** Adjusted relative risks[a] by type of synthetic oestrogen

| Type of oestrogen | Months use before first term pregnancy | Relative risk | 95% Confidence limit |
|---|---|---|---|
| E-O | Never | 1.00 | |
| | 1–12 | 1.21 | 0.50–2.93 |
| | 13–48 | 2.16 | 0.95–4.94 |
| | 48+ | 2.62 | 1.15–5.95 |
| ME | Never | 1.00 | |
| | 1–12 | 0.43 | 0.11–1.65 |
| | 13–48 | 1.46 | 0.62–3.45 |
| | 48+ | 0.57 | 0.18–1.79 |

[a]Adjusted for age at first term birth, age at menarche, menopausal status, history of benign breast disease and a family history of breast cancer.

important hormonal component; age at menarche and at first term pregnancy as well as castration appear to affect the risk. Since the risk is increasingly elevated the longer the period between menarche and first term pregnancy, exposure to endogenous oestrogens may be part of the aetiological process (Korenman, 1980). It seems entirely possible, therefore, that exogenous exposure to OCs during this period could have an effect that such exposure does not have later in life. It is clear from many studies with long term follow up that exposure to OCs later in a woman's life has no effect on breast cancer risk. Indeed in our data OC use only after first prgenancy is associated with a slight (but non-significant) protective effect.

However, since exposure at young ages, particularly before first term pregnancy, is a relatively recent practice, few data are available to evaluate any effect on breast cancer risk. OCs became widely and freely available in the UK to unmarried women in the early seventies. Such women will now only be in their thirties and early forties and will be unlikely to have accumulated long term early use more than 5 or 10 years ago. Since the formation of a palpable breast cancer may typically take the best part of twenty years, (i.e. the time for precancerous changes plus the time for a tumour to be diagnosed) it may be too soon to expect to observe a coherent epidemiological relationship (Armenian & Lilienfeld, 1979; McPherson *et al.*, 1986).

A parallel might, perhaps, be drawn with young women exposed to radiation from the atomic bombs of Hiroshima and Nagasaki who showed a dose related increase in breast cancer incidence but not until 15–20 years after exposure (Tokunaga *et al.*, 1979) or, equally, with pregnant women exposed to diethylstilboestrol who have been shown to have twice the ultimate breast cancer risk compared with unexposed controls (with a follow-up extending to forty years), but in whom no differences were observed until 22 years after exposure (Greenberg *et al.*, 1984). In the light of the evidence reported here the overall results for long term use before first pregnancy may be regarded as suggestive of an effect.

It is, therefore, important to look for clues as early as possible so that hypotheses suggested in one study as an explanation for the diverse epidemiological results, can be closely, quickly and independently investigated in others. For this reason we have drawn attention to our findings which suggest (but not strongly) a possible effect modification by synthetic oestrogen type. The data also suggest (but not strongly) a latent interval of at least ten years for E-O pills between long term early exposure and an increased risk of breast cancer diagnosis. However, the nature of these findings is such that they should be taken as hypothesis generating, as opposed to hypothesis testing. *A priori*, both

**Table X** Adjusted[a] relative risk of breast cancer associated with OC use before first term pregnancy after excluding all such use within the stated period before diagnosis (or equivalent date for controls)

| Exclusion period years X | Pill type | Accumulated months use before first term pregnancy more than X years before diagnosis | | | | $\chi^2$ | |
|---|---|---|---|---|---|---|---|
| | | *Never* | *1–12* | *13–48* | *48+* | *Trend 1 df* | *Heterogeneity 3 df* |
| 0 | All | 1.00 | 1.02 | 1.97 | 2.59 | 6.96 | 8.69 |
| | E-O | 1.00 | 1.21 | 2.16 | 2.62 | 6.93 | 7.14 |
| | ME | 1.00 | 0.43 | 1.46 | 0.57 | 0.04 | 3.31 |
| 2 | All | 1.00 | 1.17 | 1.78 | 2.46 | 6.47 | 6.75 |
| | E-O | 1.00 | 1.07 | 2.54 | 2.92 | 6.86 | 6.96 |
| | ME | 1.00 | 0.59 | 1.37 | 0.58 | 0.04 | 3.31 |
| 4 | All | 1.00 | 1.17 | 1.73 | 2.43 | 6.16 | 6.50 |
| | E-O | 1.00 | 1.13 | 2.30 | 3.09 | 7.04 | 7.14 |
| | ME | 1.00 | 0.59 | 1.37 | 0.58 | 0.04 | 3.31 |
| 6 | All | 1.00 | 1.24 | 1.83 | 2.30 | 6.28 | 6.55 |
| | E-O | 1.00 | 1.30 | 1.93 | 3.69 | 7.14 | 7.52 |
| | ME | 1.00 | 0.43 | 1.46 | 0.58 | 0.04 | 3.31 |
| 8 | All | 1.00 | 1.01 | 2.23 | 2.33 | 6.76 | 7.42 |
| | E-O | 1.00 | 0.95 | 2.69 | 4.52 | 7.71 | 7.79 |
| | ME | 1.00 | 0.59 | 1.62 | 0.63 | 0.01 | 3.90 |
| 10 | All | 1.00 | 1.20 | 2.45 | 1.65 | 5.61 | 7.93 |
| | E-O | 1.00 | 1.74 | 3.20 | 5.65 | 8.97 | 9.00 |
| | ME | 1.00 | 0.61 | 2.22 | 0.27 | 0.01 | 5.51 |
| 12 | All | 1.00 | 1.46 | 2.54 | 2.03 | 4.15 | 4.91 |
| | E-O | 1.00 | 2.15 | 3.50 | 4.20 | 6.65 | 6.70 |
| | ME | 1.00 | 1.04 | 2.34 | 0.22 | 0.25 | 3.95 |
| 14 | All | 1.00 | 1.74 | 1.37 | 1.97 | 2.25 | 2.35 |
| | E-O | 1.00 | 1.78 | 2.05 | 3.43 | 2.72 | 2.74 |
| | ME | 1.00 | 1.68 | 1.16 | 0.52 | 0.34 | 0.82 |

[a]Adjustments as before.

pill type and latency could plausibly be related to the apparent discrepancies in published epidemiological results. However, we decided to divide pill types into those containing E-O and those containing ME because this was the simplest data derived categorization we could make. We are, nonetheless, impressed by the fact that the chi square value in Table X for E-O pills did not diminish, in spite of decreasing numbers as the exclusion period increased.

If either of these hypotheses is true, then the power with which they can be reliably detected even in very large studies is currently limited. All recent studies lack adequate precision for detecting a latent effect, for in this study, for instance, only two cases and one control had been exposed for more than 4 years before first term pregnancy more than 15 years before diagnosis. Moreover pill type is probably poorly recalled in the distant past and attention to any subgroup of OCs reduces precision still further. If pill use at particular times in a woman's life, for example when anovulatory cycles are more common, is also important then such complications reduces precision yet again. It is therefore essential to pool data from different studies taking these aspects of pill use into account.

When these results are compared with those we have published previously from a similar case-control study (Vessey *et al.*, 1982) but which was negative, the following differences should be born in mind. First, recruitment to the earlier study was completed in 1980 so there was a relatively short maximum time between first OC exposure and diagnosis. Secondly, early OC use was less common in the Sixties than in the Seventies. Accordingly, long durations of OC use before first term pregnancy were much less common in the first study. In the earlier study, 13% of cases and controls under the age of 40 had used OCs before first term pregnancy, compared with 44% in this study. Moreover, of this use in the earlier study 2% was for more than a year more than ten years before diagnosis (or the equivalent date for controls) compared with 11% in this study. Finally, in the first study a larger proportion of such OC use was of ME pills, because these were more common in the 1960's.

The notion of a latent effect is consistent with the known epidemiology of breast cancer and with the observed effect of radiation and diethylstilboestrol exposure. However, the possibility of a different effect of the two synthetic oestrogens has little basis in either theory or in empirical observation. Mestranol is demethylated to E-O (Bolt *et al.*, 1974) which could give rise to lower peak plasma concentrations of active oestrogen in women taking ME containing pills than in those taking E-O containing pills (Orme *et al.*, 1983). Conceivably this could explain their lesser effect on breast cancer risk. It is just as likely, however, that the effect is a consequence of ME and E-O being correlated with some other aspect of pill composition. More endocrinological work is required, as well as observational confirmation before any action is taken on these findings. It should be noted that none of our data directly incriminate modern low dose E-O containing pills in any case.

Taking our results together with the extent of the bias that can be a consequence of plausible latent intervals (McPherson *et al.*, 1986) and a possible effect modification of pill type, it is possible that epidemiological studies are emerging with consistent results. Young women in the US apparently started using OCs around five years later than their British counterparts (Anonymous, 1985) and a much higher porportion of OCs in the CASH study contained mestranol (Sattin *et al.*, 1986).

We need to know whether these suggestions can be refuted by other well conducted studies. In particular studies can only now be begun among women with a relatively high risk of breast cancer with a substantial amount of early OC use, sufficiently long ago. While it is clear that the latency argument cannot easily explain the difference between our latest results and the study from New Zealand (Paul et al., 1986) it remains unclear whether or not some aspect of pill type or formulation might not be the explanation. The Scandinavian workers investigated a latent effect but their analyses, which seemed to show no evidence for such an effect, is not conclusive (McPherson & Drife, 1986). This is because they investigated only time since first use, which is clearly correlated with a possible latent period associated with a particular exposure, but does not measure it. Moreover, they adjusted for total OC exposure in their analysis which might adjust out a latent effect because duration of exposure likewise will be correlated with any latent period. Recent correspondence suggests that the differences observed between Norway and Sweden could be attributed to more recent early use in Norway (Ronstam & Olson, 1987).

The best way to proceed is to investigate some of these hypotheses in independent data sets, to continue with the assiduous collection of epidemiological data in which contraceptive histories are carefully recorded and, as far as possible, validated and to pool all data sets as soon as is feasible (Vessey et al., 1983). It remains quite possible that different OC formulations taken at different times in a woman's reproductive life may have quite different, but important, effects on breast cancer risk.

We would like to thank Mrs M.S. Simmonds, Mrs E.H. Hilton, Mrs J. Young, Mrs A. Bateman and Mrs M. McArthur for interviewing the patients and the consultants at the participating hospitals for allowing us to include patients under their care. The Imperial Cancer Research Fund kindly provided financial support. Mrs Anne Reeve for patient and assiduous secretarial work.

### References

ANONYMOUS (1985). Another look at the pill and breast cancer. Editorial. *Lancet*, **ii**, 985

ARMENIAN, H.K. & LILIENFELD, A.M. (1983). Incubation periods of disease. *Epidemiol. Rev.*, **5**, 1.

BOLT, H.M. & BOLT, W.H. (1974). Pharmokinetics of menstranol in man in relation to its oestrogen activity. *Eur. J. Clin. Pharmacol.*, **7**, 295.

BONE, M. (1979). The family planning services: Changes and effects. *Office of Population Census and Surveys*. HM Stationery Office: London.

BRESLOW, N. & DAY, N.E. (1980). The analysis of case-control studies. *IARC*.

COULTER, A., VESSEY, M., McPHERSON, K. & CROSSLEY, B. (1986). The ability of women to recall their oral contraceptive histories. *Contraception*, **33**, 127.

DAY, N.E. (1984). Epidemiological data and multi-stage carcinogenesis. *IARC Sci. Publ.*, **56**.

DRIFE, J.O. (1981). Breast cancer, pregnancy and the pill. *Br. Med. J.*, **283**, 778.

GREENBERG, E.R., BARNES, A.B., RESSEGUIE, L. & 7 others (1984). Breast cancer in mothers given diethylstibestrol in pregnancy. *New Engl. Med. J.*, **311**, 1393.

HARRIS, N.V., WEISS, N.S., FRANCIS, A.M. & POLISSER, L. (1982). Breast cancer in relation to patterns of oral contraceptive use. *American Journal Epidemiology*, **116**, 643.

KAY, C. (1986). *Steroidal Contraceptive Pilot Study Report*. Royal College of General Practitioners: London.

KORENMAN, S.G. (1980). Oestrogen window hypothesis of the aetiology of breast cancer. *Lancet*, **i**, 700.

McMAHON, B., COLE, P. & BROWN, J. (1973). Etiology of human breast cancer. A review. *J. Natl Cancer Inst.*, **50**, 21.

McPHERSON, K., NEIL, A. VESSEY, M.P. & DOLL, R. (1983). Oral contraceptives and breast cancer. *Lancet*, **ii**, 1414.

McPHERSON, K. & DRIFE, J.O. (1986). The pill and breast cancer: Why the uncertainty? Editorial. *Br. Med. J.*, **293**, 709.

McPHERSON, K. COOPE, P.A. & VESSEY, M.P. (1986). Early oral contraceptive use and breast cancer – theoretical effects of latency. *Br. J. Epidemiol. Comm. Hlth.*, **40**, 289.

MANT, D., VESSEY, M.P., NEIL, A., McPHERSON, K. & JONES, L. (1987). Breast self examination and breast cancer stage at diagnosis. *Br. J. Cancer*, **55**, 207.

MEIRIK, O., LUND, E., ADAMI, H.-O., BERGSTROM, R. CHRISTOFFEN, T. & BERGSJO, P. (1986). Oral contraceptive use and breast cancer in young women. *Lancet*, **ii**, 650.

MILLER, D.R., ROSENBERG, L., KAUFMAN, D.W., SCHOTHENFELD, D., STOLLEY, P.D. & SHAPIRO, S. (1986). Breast cancer risk in relation to early oral contraceptive use. *Obst. Gynaecol.*, **68**, 863.

OLSON, H., OLSON, M.L., MOLLER, T.R., RONSTEN, J. & HOLM, P. (1985). Oral contraceptive use and breast cancer in young women in Sweden. *Lancet*, **ii**, 748.

OPCS. (1982). OPCS Monitor MBI 85/2. Cancer Registration.

ORME, M.L.E., BOCK, D.J. & BRECKENRIDGE, A.M. (1983). Clinical pharmacokinetics of oral contraceptive steroids. *Clin. Pharmacokinetics*, **8**, 95.

PAFFENBERGER, R.S., FASAL, E., SIMMONS, M.E. & KAMBERT, J.B. (1977). Cancer risk as related to use of oral contraceptives during the fertile years. *Cancer*, **39**, 1887.

PAUL, C., SKEGG, D.G., SPEARS, G.F.S. & GALDER, J.M. (1986). Oral contraceptives and breast cancer: A national study. *Br. Med. J.*, **293**, 723.

PAUL, C., SKEGG, D. & SPEARS, G. (1986). The pill and breast cancer: Why the uncertainty. *Br. Med. J.*, **293**, 1432.

PIKE, M.C., HENDERSON, B.E., CASAGRANDE, J.J., ROSARIO, I. & GRAY, G.E. (1981). Oral contraceptive use and early abortion as risk factors for breast cancer in young women. *Br. J. Cancer*, **43**, 72.

PIKE, M.C., HENDERSON, B.E., KRAILO, M.D., DUKE, A. & ROY, S. (1983). Breast cancer in young women and oral contraception: A possible modifying effect of formulation and age at use. *Lancet*, **ii**, 926.

RONSTAM, J. & OLSSON, H. (1987). Oral contraceptives and breast cancer. *Lancet*, **i**, 636.

ROSENBERG, L., MILLER, D.R., KAUFMAN, D.W. & 4 others (1984). Breast cancer and oral contraceptive use. *Am. J. Epidemiol.*, **119**, 167.

SATTIN, R.W., ROBIN, G.L., WINGO, P.A., WEBSTER, L.A. & ORY, H.W. (1986). Oral contraceptive use and the risk of breast cancer: The Cancer and Steroid Hormone Study of the Centre for Disease Control and the National Institute of Child Health and Human Development. *New Engl. J. Med.*, **313**, 405.

STADEL, B.V., RUBIN, G.L., WEBSTER, L., SCHLESSELMAN, J.J. & WINGO, P.A. (1985). Oral contraceptives and breast cancer in young women. *Lancet*, **ii**, 970.

TOKUNAGA, M., NORMAN, J.E. & ASANO, M. (1979). Malignant breast tumours among atomic bomb survivors, Hiroshima and Nagasaki. *J. Natl Cancer Inst.*, **62**, 1347.

VESSEY, M.P., McPHERSON, K., YEATES, D. & DOLL, R. (1982). Oral contraceptive use and abortion prior to first term pregnancy in relation to breast cancer risk. *Br. J. Cancer*, **45**, 327.

VESSEY, M.P., BARON, J., DOLL, R., McPHERSON, K. & YEATES, D. (1983). Oral contraceptive and breast cancer: Final report of an epidemiological study. *Br. J. Cancer*, **47**, 455.

VESSEY, M.P., McPHERSON, K., PETO, J. & PIKE, M.C. (1983). Oral contraceptives and breast cancer. *Lancet*, **ii**, 1019.